# The Impact of Cognitive, Social and Physical Limitations on Income in Community Dwelling Adults With Chronic Medical and Mental Disorders

**DOI:** 10.5539/gjhs.v7n5p183

**Published:** 2015-02-24

**Authors:** Clara E. Dismuke, Leonard E. Egede

**Affiliations:** 1 Health Equity and Rural Outreach Innovation Center, Ralph H. Johnson Veterans Affairs Medical Center, Charleston, SC, USA; 2 Center for Health Disparities Research and Division of General Internal Medicine & Geriatrics, Medical University of South Carolina, Charleston, SC, USA

**Keywords:** chronic disease, cognitive, social, physical limitations, income, Heckman

## Abstract

**Introduction:**

As much as 45% of the US population has at least one chronic condition while 21% have multiple chronic diseases. The study examined the impact of cognitive, social and physical limitations on the personal income of U.S. adults with seven chronic diseases.

**Methods:**

A cross-sectional analysis of 19,357 US adults with seven chronic diseases (diabetes, hypertension, heart disease, stroke, depression, emphysema and joint disease) from the 2006 Medical Expenditure Panel Survey (MEPS) was performed. The effect of seven chronic diseases and their associated cognitive, social, and physical limitations on personal income was assessed using a two-stage Heckman model.

**Results:**

Depression emerged as the only chronic disease that was independently associated with a significant $1,914 decrease in personal income (95% CI -$2,938--$890). Social and cognitive limitations resulted in $1,944 (95% CI -$3,378--$511) and $3,039 (95% CI -$4,418-$1,659) decreases in personal incomes respectively while physical limitations did not result in a statistically significant reduction. Being Non-Hispanic Black, Hispanic, Other Race, female, never married, married, less than a bachelor’s degree, publicly insured, uninsured, or having a health status less than very good were also associated with significant reductions in personal income.

**Conclusions:**

The findings of this study suggest a need to determine the specific limitations associated with common chronic diseases and identify appropriate compensatory strategies to reduce their impact on income.

## 1. Introduction

Recent reports suggest that as much as 45% of the US population has at least one chronic condition while a staggering 21% have multiple chronic diseases (Anderson & Horvath, 2004). Among the most prevalent chronic diseases are diabetes, hypertension, heart disease, stroke, chronic respiratory diseases, and obesity. It is generally believed that the number of individuals with chronic diseases will continue to increase for the next 30 years (Anderson & Horvath, 2004).

One of the many consequences of chronic diseases is decreased employment and reductions in income. Studies show that a significant percentage of individuals with disabilities participate in the workforce, however, many earn substantially less than individuals without disabilities (Randolf, 2004; U.S. Census Bureau, 2010; Findley et al., 2004; Ng et al., 2001; Yelin et al., 2003; National Academy on an Aging Society, 2003; Brown et al., 2006; Wehman et al., 2003; Krause & Terza, 2006; U.S. Centers For Disease Control, 2003; Yelin et al., 2007; Finkelstein et al., 2005). Compounding these income disparities are differences in income that are associated with gender and race/ethnicity. Data from the 1994 Survey of Income and Program Participation Survey (SIPP) indicate that men with no disability earned 49% more than women with no disability (Jans et al., 1999). Men with non-severe disabilities earned 55% more than women with non-severe disabilities and women with non-severe disabilities had the lowest income overall. Similarly, racial/ethnic disparities in income have been reported for both disabled and non-disabled populations (U.S. Census Bureau, 2007 & 2009).

A final interrelated factor associated with income reductions in chronic diseases is the degree to which cognitive, physical and social limitations associated with the condition impact income. Many chronic diseases result in permanent cognitive (attention, memory, communication issues) and physical (sensory deficits, hemiplegia, ambulation changes) limitations which result in reductions in social interactions or “social limitations”. Consequently, individuals with chronic diseases and associated cognitive, physical, and social limitations are likely to be at higher risk of experiencing work-related/employment difficulties and decreased income. Indeed, severe psychiatric disorders have been shown to be associated with a 52% increase in poverty depth and 3.10 times the odds of being poor (Vick et al., 2012). Rheumatoid Arthritis has also been shown to be associated with lower annual earnings (Sullivan et al., 2010). However, few studies have attempted to measure the effect of cognitive, physical and social limitations on income across a number of common chronic diseases. Therefore, the primary aim of this study was to examine the effect of chronic disease on the total personal income of U.S. adults 18 years of age and older. A secondary aim was to examine the additional effect of cognitive, physical and social limitations on the total personal income of U.S. adults with seven common chronic diseases.

For this study we used data from the 2006 Medical Expenditure Panel Survey (MEPS) to determine the effect of seven chronic diseases along with associated cognitive, physical and social limitations on total personal income. We tested the hypothesis that health limitations diminish “human capital”, resulting in diminished work capacity, productivity, and income. The concept of human capital emerges from a human capital framework where human capital is defined as physical and intellectual attributes that enable a person to earn income during a work life (Gamboa et al., 2006). The human capital framework recognizes that individuals with limitations may acquire disability capital, which is training that is unique to individuals with specific types of limitations (Charles, 2003), however it is not well known how different types of limitations associated with chronic diseases affect income. We hypothesized that individuals with chronic diseases would have significantly lower total personal income compared to those without chronic diseases after adjusting for relevant confounding factors. We also hypothesized that individuals with cognitive, physical, and social limitations would have even greater reductions in total personal income when compared to those without these limitations after adjusting for chronic diseases and relevant confounders.

## 2. Methods

### 2.1 U.S. Sample

We analyzed data from all respondents to the MEPS Household Component survey for 2006 that were older than 17 years. The MEPS is cosponsored by the Agency for Healthcare Research and Quality (AHRQ) and the National Center for Health Statistics (Agency for Healthcare Research and Quality, 2006 & 2008). The MEPS sample is drawn from reporting units in the previous year’s National Health Interview Survey and is a nationally representative sample (with oversampling for Blacks and Hispanics) of the US civilian non-institutionalized population. The household component collects detailed information for each person in the household on demographic characteristics, health conditions, health status, medical services utilization, charges and source of payments, access to care, satisfaction with care, health insurance coverage, income and employment.

### 2.2 Study Variables: All Variables Were Based on Self-Report

#### 2.2.1 Income

Personal income was defined as individual income from all sources in 2006 including wage, business, unemployment compensation, workers compensation, interest, dividend, sales, pension, Social Security, Trust/Rent, Veteran Administration, Investment Retirement Account (IRA), Refund, Alimony, Child Support, other regular cash contribution, SSI, public assistance and other income.

#### 2.2.2 Cognitive, Physical and Social Limitations

Limitations were defined as any cognitive, social or physical limitations based on self-report. Specifically, cognitive limitation was defined as a yes response to: “Do any of the adults in the family 1) Experience confusion or memory loss such that it interferes with daily activities? 2) Have problems making decisions to the point that it interferes with daily activities? 3) Require supervision for their own safety?” Social limitation was defined as a yes response to: “Is anyone in the family limited in participating in social, recreational or family activities because of an impairment or a physical or mental health problem?” Physical limitation was defined as a yes response to: “Does anyone in the family have difficulties walking, climbing stairs, grasping objects, reaching overhead, lifting, bending or stooping, or standing for long periods of time?” For survey respondent households with more than one member, the individual with the limitation was identified by the responder (owner or renter of dwelling) or their proxy if the responder was not present (Agency for Healthcare Research and Quality 2006, 2008).

#### 2.2.3 Chronic Diseases

Presence of at least one of seven chronic diseases was based on a yes response to the following question: “Has the person ever been told by a health professional that the person has: diabetes (except during pregnancy), hypertension, heart disease (coronary heart disease, heart attack/myocardial infarction, angina, other heart disease), stroke or transient ischemic attack (mini stroke), emphysema and joint disease (joint pain, arthritis). Depression was defined as a score of ≥3 on the 2-Item Patient Health Questionnaire (PHQ-2) (Kroenke et al., 2003). The PHQ-2 was designed to inquire about depressed mood and anhedonia over the past 2 weeks, Scoring ranges from 0-6 with 0 indicating (“not at all”) to 3 (“nearly every day”). The PHQ-2 has 83% sensitivity and 92% specificity for identifying major depression (Kroenke et al., 2003).

#### 2.2.4 Demographic and Clinical Variables

Age was based on the following age groups: 18-24 years, 25-44 years, 45-64 years, and 65-85 years. We included working individuals ≥65 years because the US Census Bureau notes that 20% of American males and 11% of American females over the age of 65 were in the labor force in 2008. Race/ethnicity was categorized as Non-Hispanic White, non-Hispanic Black, Hispanic, and Other Race. Marital status was categorized as married, never married, and widowed/divorced/separated. Education was categorized as less than high school graduate, high school graduate, less than bachelor’s degree, college graduate or greater. Perceived health status was categorized as excellent, very good, good, fair and poor. Insurance status was categorized as private health insurance, public health insurance and uninsured. Body Mass Index (BMI) was measured in categories: underweight = BMI < 18.5, normal weight = BMI ≥18.5 & ≤24.9, overweight = BMI ≥25.0 & ≤29.9, and obesity = BMI ≥30.0.

### 2.3 Analysis

For this study we performed four types of analyses. First, we calculated weighted proportion and confidence intervals for the key demographic characteristics of the sample. Second, we calculated unadjusted mean personal income for the variables included in the personal income model. Third, we used the two-stage Heckman procedure to estimate the personal income model while controlling for income reporting bias. Heckman’s model (1979) provides the most common method of addressing such biases by estimating determinants of the labor-market participation decision (i.e. probability of having any personal income) and then to use those estimates to construct an Inverse Mills Ratio, which is added to the second stage of the personal income equation. Fourth, we calculated the estimated adjusted mean personal income and confidence intervals for key demographic characteristics of the sample. We used STATA V10 for all analyses to account for the complex survey design of MEPS (Agency for Healthcare Research and Quality, 2006 & 2008).

## 3. Results

The MEPS sample for 2006 included 19,357 U.S. adults 18 years and older. Table 1 reports the percentages of U.S. adults in the sample with any of seven chronic diseases and three functional limitations along with their demographic characteristics. For chronic diseases, 7.8% had diabetes, 26.8% hypertension, 9.6% heart disease, 2.3% stroke, 8.7% depression, 1.3% emphysema and 39.0% joint disease. With respect to functional limitations, 4.4% had cognitive, 12.2% physical and 5.2% social limitations. The most frequent age group was age 25-44 (37.8%) and the least frequent was age 18-24 (12.2%). Males comprised 48% of the sample while 69.4% were Non-Hispanic White, 11.1% Non-Hispanic Black, 13.0% Hispanic and 6.4% Non-Hispanic Other Race. Married adults comprised 54.9% of the sample while 24.8% were never married and 20.3% were no longer married. U.S. adults with less than a high school education comprised less than 17.9% of the sample, while 31.1% had a high school education, 23.6% less than a college education and 27.4% with a college education or higher. U.S. adults who considered themselves to have excellent health status comprised 24.7% of the sample, while 34.6% considered themselves to have very good, 28.4% good, 9.2% fair and 3.2% poor health status. U.S. adults with any private health insurance comprised 71.7% of the sample while 13.8% had public health insurance only, and 14.5% were uninsured. U.S. adults with a BMI <18.5 comprised 1.7% of the sample while 35.7% had a BMI of 18.5-24.9, 35% a BMI of 25.0-29.9, and 27.5% a BMI ≥30.

**Table 1:** Clinical and socioeconomic characteristics of the U.S. sample, 2006

Variable	n = 19,357 % Effected	95% Confidence Interval
Diabetes	7.8	(7.3,8.3)
Hypertension	26.8	(25.8,27.7)
Heart Disease	9.6	(9.1,10.2)
Stroke	2.3	(2.0,2.5)
Depression (PHQ2)	8.7	(8.2,9.2)
Emphysema	1.3	(1.1,1.5)
Joint Disease	39.0	(37.7,40.3)
Cognitive Limitation	4.4	(4.0,4.8)
Physical Limitation	12.2	(12.5,13.0)
Social Limitation	5.2	(4.7,5.6)
Age 18-24	12.2	(11.5,12.8)
Age 25-44	37.8	(36.6,38.9)
Age 45-64	34.0	(33.0,35.0)
Age 65-85	16.1	(15.3,16.9)
Male	48.0	(47.4,48.7)
White/Not Hispanic	69.4	(67.6,71.2)
Black/Not Hispanic	11.1	(10.0,12.1)
Hispanic	13.0	(11.8,14.2)
Others/Not Hispanic	6.4	(5.6,7.3)
Married	54.9	(53.8,56.0)
Never Married	24.8	(23.8,25.7)
Widowed/Divorced/Separated	20.3	(19.4,21.1)
Less Than High School	17.9	(17.0,18.7)
High School	31.1	(30.1,32.2)
Less Than Bachelor	23.6	(22.8,24.4)
College or Greater	27.4	(26.0,28.7)
Excellent Health Status	24.7	(23.7,25.6)
Very Good Health Status	34.6	(33.7,35.5)
Good Health Status	28.4	(27.5,29.3)
Fair Health Status	9.2	(8.6,9.7)
Poor Health Status	3.2	(2.8,3.5)
Any Private Health Insurance	71.7	(70.6,72.8)
Public Health Insurance Only	13.8	(13.0,14.6)
Uninsured	14.5	(14.0,15.3)
BMI <18.5	1.7	(1.5,2.0)
BMI 18.5 - 24.9	35.7	(34.6,36.6)
BMI 25.0 - 29.9	35.0	(34.2,35.8)
BMI ≥30.0	27.5	(26.7,28.4)

Table 2 provides the unadjusted mean personal income by chronic disease, limitation, and demographic characteristics. The unadjusted mean personal income for the full sample was $33,657. For individuals with chronic diseases, the unadjusted mean was $27,947 for those with diabetes, $32,402 for hypertension, $33,963 for heart disease, $26,488 for stroke, $20,799 for depression, $21,681 for emphysema and $33,276 for joint disease. For functional limitations, the unadjusted mean personal income for physical was $23,387, $19,166 for social and $16,344 for cognitive limitations. Unadjusted mean personal income increased with age category until retirement age, was higher among males, Non-Hispanic Whites, Married, and increased with education level, health and private insurance status and BMI until the level of obesity.

**Table 2:** Unadjusted mean personal income for the U.S. sample, 2006 (n = 19,357).

Variable	Unadjusted Mean Personal Income	95% Confidence Interval
Full Sample	$33,657	($32,869,$34,444)
Diabetes	$27,947	($26,087,$29,807)
Hypertension	$32,402	($31,181,$33,623)
Heart Disease	$33,963	($33,148,$34,777)
Stroke	$26,488	($23,477,$29,500)
Depression (PHQ2)	$20,799	($19,428,$22,170)
Emphysema	$21,681	($17,585,$25,777)
Joint Disease	$33,276	($32,250,$34,302)
Cognitive Limitation	$16,344	($15,066,$17,622)
Physical Limitation	$23,387	($21,978,$24,796)
Social Limitation	$19,166	($17,476,$20,856)
Age 18-24	$12,888	($12,061,$13,716)
Age 25-44	$36,849	($35,512,$38,186)
Age 45-64	$40,478	($39,202,$41,754)
Age 65-85	$27,472	($26,093,$28,852)
Male	$38,900	($37,908,$39,983)
Female	$28,807	($27,948,$29,666)
White/Not Hispanic	$36,987	($36,006,$37,968)
Black/Not Hispanic	$25,240	($23,975,$26,505)
Hispanic	$22,707	($21,572,$23,843)
Others/Not Hispanic	$34,367	($31,652,$37,082)
Married	$38,115	($37,065,$39,165)
Never Married	$24,023	($22,794,$25,253)
Widowed/Divorced/Separated	$33,357	($31,942,$34,773)
Less Than High School	$16,046	($15,338,$16,755)
High School	$26,585	($25,806,$27,364)
Less Than Bachelor	$32,918	($31,777,$34,059)
College or Greater	$53,873	($52,301,$55,445)
Excellent Health Status	$39,218	($37,512,$40,924)
Very Good Health Status	$36,754	($36,656,$37,853)
Good Health Status	$30,692	($29,526,$31,857)
Fair Health Status	$22,002	($20,624,$23,379)
Poor Health Status	$16,841	($15,114,$18,568)
Any Private Health Insurance	$40,245	($39,322,$41,168)
Public Health Insurance Only	$15,113	($14,229,$15,997)
Uninsured	$18,683	($17,747,$19,619)
BMI < 18.5	$23,633	($20,177,$27,088)
BMI 18.5 - 24.9	$33,080	($31,834,$34,326)
BMI 25.0 - 29.9	$35,963	($34,875,$37,052)
BMI ≥30.0	$32,104	($31,023,$33,185)

Table 3 shows the results of the Heckman two-step model for income. Being male, greater than age 25 (relative to 18-25), widowed/divorced/separated (relative to married) and each additional year of education was associated with a greater probability of having any personal income. When probability of having any personal income was incorporated into the income model (2^nd^ stage of the model) depression (-$1,914), cognitive limitation (-$3,039), and social limitation (-$1,944) were independently associated with decreased personal income. Compared with age 18-25, age 25-44 ($12,030), 45-64 ($13,487) and 65-85 ($7,942) were associated with increased personal income. Compared to married, widowed/divorced/single ($3,206) was associated with increased personal income. Compared with less than high school, high school ($6,831), less than college education ($10,718) and college education or greater ($20,828) were associated with increased personal income. Compared with being Non-Hispanic White, being Non-Hispanic Black (-$3,157), Hispanic (-$3,213) and Non-Hispanic Other Race (-$1,753) were associated with decreased personal income. Compared to married, being never married (-$1,044) was associated with decreased personal income. Compared to excellent health status, good health ($-1,746), fair (-$4,623) and poor (-$2,237) were associated with decreased personal income. Compared to private insurance, public only (-$9,577) and uninsured (-$10,430) were associated with decreased personal income.

**Table 3:** Heckman’s two-step model of personal income for the U.S. sample, 2006 (n = 19,357).

Variable	Coefficient Estimate	Standard Error	P-Value	95% Confidence Interval
**Personal Income Equation**		
Diabetes	-$489	$706.70	0.489	(-$1,881,$902)
Hypertension	$342	$478.41	0.475	(-$599,$1,284)
Heart Disease	-$327	$751.39	0.663	(-$1,807,$1,152)
Stroke	$1,694	$1,086.50	0.120	(-$445,$3,834)
Depression (PHQ2)	-$1,914	$520.01	<0.001	(-$2,938,-$890)
Emphysema	-$1,784	$1,936.06	0.357	(-$5,598,$2,028)
Joint Disease	$414	$319.33	0.195	(-$214,$1,043)
Cognitive Limitation	-$3,039	$700.27	<0.001	(-$4,418,$1,659)
Physical Limitation	-$1,353	$755.03	0.074	(-$2,840,$133)
Social Limitation	-$1,944	$727.82	0.008	(-$3,378,-511)
Age 25-44*	$12,030	$21.57	<0.001	($11,988,$12,072)
Age 45-64*	$13,487	$25.41	<0.001	($13,437,$13,537)
Age 65-85*	$7,942	$18.69	<0.001	($7,905,$7,979)
Male*	$7,882	$12.32	<0.001	($7,858,$7,907)
Black/Not Hispanic*	-$3,157	$8.99	<0.001	(-$3,174, -$3,139)
Hispanic*	-$3,213	$5.76	<0.001	(-$3,225, -$3,202)
Others/Not Hispanic*	-$1,753	$3.02	<0.001	(-$1,759, -$1,747)
Never Married*	-$1,044	$5.07	0.003	(-$1,054, -$1,034)
Widowed/Divorced/Separated*	$3,206	$4.04	<0.001	($3,198,$3,214)
High School*	$6,831	$2.08	<0.001	($6,827,$6,835)
Less Than College*	$10,718	$2.08	<0.001	($10,714,$10,722)
College or Greater*	$20,828	$2.08	<0.001	($20,823,$20,832)
Very Good Health Status	-$848	$437.54	0.053	(-$1,711,$12)
Good Health Status	-$1,746	$616.83	0.005	(-$2,961,-$531)
Fair Health Status	-$4,623	$758.73	<0.001	(-$6,118,-$3,129)
Poor Health Status	-$2,237	$914.52	<0.001	(-$8,442,-$4,840)
Public Health Insurance Only	-$9,577	$19.86	<0.001	(-$9,616,-$9,538)
Uninsured	-$10,430	$21.49	<0.001	(-$10,472,-$10,388)
BMI 18.5 - 24.9	$2,334	$1,410.48	0.099	(-$443,$5,112)
BMI 25.0 - 29.9	$2,561	$1,412.82	0.071	(-$220,$5,344)
BMI ≥30.0	$2,237	$1,426.96	0.118	(-$573,$5,047)

**Probability of Any Personal Income**		
Male	0.3644	0.022	<0.001	(0.320,0.407)
Age 25-44	0.637	0.035	<0.001	(0.567,0.706)
Age 45-64	0.750	0.038	<0.001	(0.675,0.824)
Age 65-85	0.551	0.050	<0.001	(0.453,0.650)
Black/Not Hispanic	-0.265	0.030	<0.001	(-0.324,-0.207)
Hispanic	-0.170	0.033	<0.001	(-0.235,-0.105)
Others/Not Hispanic	-0.089	0.048	0.063	(-0.183,0.005)
Never Married	-0.150	0.034	<0.001	(-0.216,-0.083)
Widowed/Divorced/Single	0.119	0.035	0.001	(0.0450,0.189)
Education In Years	0.061	0.003	<0.001	(0.055,0.067)
Public Health Insurance Only	-0.586	0.032	<0.001	(-0.649,-0.523)
Uninsured	-0.634	0.031	<0.001	(-0.696,-0.573)
Constant Term	-0.061	0.052	0.240	(-0.163,0.041)
Model Statistics		
rho	0.998	0.001		(0.996,0.999)
sigma	2,9470	484.72		(28,530,30,440)
lambda	2,9423	489.80		(28,458,30,388)

* Adjusted Coefficients to Account for Variables in Both Estimation and Selection Models Using: Adjusted Coefficient = estimation coefficient-(selection coefficient*e(rho)*e(sigma)*delta) where delta = lambda*(lambda*selection prediction)

Table 4 shows the adjusted mean personal income after controlling for the probability of having any income and covariates. For chronic diseases and functional limitations, compared to unadjusted personal income, adjusted personal income significantly decreased by 19.2% for depression ($16,807 vs 20,799, 14.4% for cognitive limitations ($13,989 vs $16,344) and 13.1% for social limitations ($16,665 vs $19,166).

**Table 4:** Adjusted mean personal income for the U.S. sample, 2006 (n = 19,357).

Variable	Adjusted Mean Personal Income	95% Confidence Interval
Diabetes	$23,467	($22,153,$24,780)
Hypertension	$27,537	($26,719,$28,355)
Heart Disease	$26,450	($25,128,$27,772)
Stroke	$21,851	($19,762,$23,940)
Depression (PHQ2)	$16,807	($15,784,$17,830)
Emphysema	$19,568	($15,839,$23,296)
Joint Disease	$28,396	($27,765,$29,027)
Cognitive Limitation	$13,989	($12,782,$15,196)
Physical Limitation	$20,094	($19,051,$21,136)
Social Limitation	$16,665	($15,436,$17,894)
Age 18-24	$4,682	($3,529,$5,834)
Age 25-44	$30,306	($29,324,$31,288)
Age 45-64	$35,234	($34,185,$36,282)
Age 65-85	$22,891	($21,692,$24,090)
Male	$34,322	($33,576,$35,069)
Female	$22,182	($21,606,$22,758)
White/Not Hispanic	$34,416	($33,869,$35,143)
Black/Not Hispanic	$20,649	($19,574,$21,724)
Hispanic	$15,667	($14,616,$16,719)
Others/Not Hispanic	$30,824	($28,484,$33,163)
Married	$32,654	($31,888,$33,420)
Never Married	$16,358	($15,334,$17,372)
Widowed/Divorced/Separated	$27,623	($26,463,$28,784)
Less Than High School.	$10,527	($9,764,$11,290)
High School	$25,345	($24,642,$26,047)
Less Than College	$31,903	($31,008,$32,798)
College or Greater	$47,772	($46,156,$49,388)
Excellent Health Status	$32,185	($31,183,$33,187)
Very Good Health Status	$31,282	($30,559,$32,006)
Good Health Status	$26,235	($25,458,$27,013)
Fair Health Status	$18,142	($17,052,$19,232)
Poor Health Status	$13,470	($11,957,$14,984)
Any Private Health Insurance	$37,922	($37,178,$38,666)
Public Health Insurance Only	$9,688	($8,785,$10,591)
Uninsured	$12,637	($11,581,$13,693)
BMI < 18.5	$16,608	($13,901,$19,316)
BMI 18.5 - 24.9	$27,245	($26,562,$27,928)

## 4. Discussion

We examined data from 19,357 adults who responded to the 2006 MEPS to determine the impact of chronic disease and cognitive, physical, and social limitations on personal income. Two key findings emerged from this study. First, after adjusting for all relevant confounding factors, depression was the only chronic condition that was independently associated with decreased personal income. Second, among individuals with chronic diseases and limitations, individuals with cognitive and social limitations earned substantially less personal income than individuals without limitations. Our findings offer new and compelling evidence regarding the effect of chronic disease and subsequent limitations on personal income.

In this study we proposed that individuals with chronic diseases would experience reduced “human capital” thereby resulting in a reduced capacity for work productivity and subsequently reduced personal income (Gamboa et al., 2006). However, after controlling for relevant confounding factors, depression was the only chronic condition that statistically significantly reduced personal income. Depressed individuals earned approximately $1,914 less than non-depressed individuals which exceeded a non-significant differential among individuals with stroke. Given the range of sequela commonly associated with stroke, it is surprising that depression resulted in a greater reduction in personal income.

The findings observed in this study are consistent with the results from previous studies. In a study of more than 30,000 adults who participated in the 1999 National Health Interview Survey, Egede (2007) found that among individuals with common chronic diseases (hypertension, diabetes mellitus, coronary artery disease, congestive heart failure, stroke, chronic obstructive pulmonary disease, and end-stage renal disease) who were employed, 16% were absent from work more than 6 days in the past year and approximately 50% had missed at least 1 day due to illness. Egede concluded that the reported missed workdays and days spent in bed represented both economic losses to the individual and society. Egede also noted that individuals with major depression experienced even greater work-related productivity loss in addition to greater health resource utilization and higher degrees of functional disability. The cost of missed workdays and overall reductions in work productivity are staggering.

Similarly, Stewart and colleagues (2003) examined data from the American Productivity Audit (2001-2002) and estimated that US workers with depression who were employed the previous week experienced the equivalent of $44 billion per year in “lost productive time” which exceeded their non-depressed peers by $31 billion. They also concluded that unfortunately much of the lost productive time due to depression is not visible to employers and is explained by reduced work performance. These conclusions are supported by Adler and colleagues who noted that depression can negatively influence multiple dimensions of job performance including mental-interpersonal issues, managing time, and overall performance output (Adler et al., 2006). Collectively, these employee performance issues are at the expense of the employer. Therefore, the treatment of individuals with depression appears to reduce the economic cost in that increased work productivity and reductions in lost work days have been reported among individuals receiving treatment for depression (Zhang et al., 1999).

Our second major finding was the type of limitation that individuals with chronic diseases experience can have a substantial differential effect on personal income. The results of this study indicate that a large number of adults with chronic disease and associated limitations participate in the workforce as noted in previous reports (Findley et al., 2004; Findley & Sambamoorthi, 2003). However, participating in the workforce among individuals with limitations occurs at a cost of reduced wages. After adjustments, individuals with cognitive limitations earned over $3000 less than individuals without cognitive limitations while individuals with social limitations earned over $1900 less than their counterparts who did not report social limitations.

Several previous studies have reported a differential effect of limitations on personal income among individuals with a range of disabilities (Randolph, 2004; Ng et al., 2001; National Academy on Aging Society, 2003). Specifically, those individuals with physical limitations earned more than $1300 less than adults without physical limitations although this value was not statistically significant. These findings collectively are supported by an early study of disability and employment by Greenwood and colleagues which showed that employers viewed potential employee and current employees with physical disabilities more favorably than those with mental, emotional or communication disabilities in multiple areas including recruitment, selection, acceptance and performance expectation (Greenwood et al., 1991). They found that employers responded differently to individuals with disabilities based on the type of disability in that they were more willing to hire and subsequently support an individual with a physical disability compared to an employee with an mental (i.e. cognitive), emotional, or communication disability.

Consequently, the type of limitation or disability has a differential effect on personal income. For example, Kaye (2009) found that individuals with physical disabilities were not significantly underrepresented in occupations requiring physical skills in handling objects. In contrast, individuals with cognitive disabilities were not overrepresented in such positions.

However, the presence of a cognitive limitation appears to have a substantially greater impact on personal income than either social or physical limitations. One possible explanation could be that fewer established mechanisms currently exist to compensate for cognitive limitations than exist of physical limitations. For example, community-based modifications for individuals with physical disabilities currently exist in the form of ramps, door modifications, bathroom modifications, and specific ergonomic modifications for the workplace. However, fewer modifications exist for individuals with cognitive limitations and those that do exist are primarily geared to be managed by the individual with the disability (i.e. electronic devices for memory) thereby decreasing the likelihood of success. This explanation would rule out age-related cognitive limitations as a primary explanation because age was controlled in this study for such differences.

Little if any current information currently exists regarding the impact of social limitations on personal income. We believe these findings offer insights into how social limitations might impact personal income. We would hypothesize that the individuals in the study would conceptualize social limitations and reductions in engagements with work-related peers. In the workplace, networking plays a critical role in work and career success as individuals with good networks are more likely to be connected individuals who make decisions about the workplace and determine policy and direction. We would then hypothesize that individuals that are not connected to such networks would be less likely to be engaged in administrative roles or connected to such individuals and consequently be lower earners in the workplace. This hypothesis is supported by Kaye (2009) who completed a survey of the US population to identify occupational and individual factors that influence representation of workers with disabilities across a range of occupations. Kaye found that workers with disabilities were disproportionately employed in entry-level positions or underemployed in low wage positions. Kaye’s study suggested that employer discrimination and low expectations of individuals with disabilities contributed to the occupational and personal income disparities. In addition, the study concluded that individuals with disabilities were significantly underrepresented in occupations that required proficiency in communicating with individuals outside of the organization. It would appear that this factor in itself would create a social limitation that reduces personal income as a result of limited inter-organization contact. Further study is necessary to adequately test this hypothesis.

The results of this study have at least two policy implications. First, although a substantial number of individuals with disabilities are employed, their opportunities for advancement and subsequently higher personal income appear limited. According to Kaye (2009), workers with disabilities are not employed to their highest potential which translates into lower wage positions. The findings reported by Kaye did not delineate the exact cause of whether such disparities were due to employer discrimination or worker reluctance to actively pursue better paying, higher intellectually demanding jobs. Schur (2003) suggests that employees with disabilities may be content working in lower wage or part-time jobs because otherwise they would not be employed. However, the consequences of such choices are fewer benefits and higher poverty rates. In many cases, workers with disabilities who choose to work in low wage positions are left without benefits completely because of continued increases in healthcare costs which have forced many employers to increase premiums for employees or eliminate coverage altogether (Emanuel, 2008). For individuals with chronic diseases, the absence of healthcare coverage results in many going into debt in attempts to pay medical bills (Mitka, 2008). Ultimately, some will choose to reduce or discontinue care which will eventually result in greater costs over time.

Second, we found being uninsured had an even more detrimental effect on personal income than did having depression or a cognitive or social limitation alone. After controlling for other covariates including chronic disease and functional limitations, mean personal income for the uninsured declined by 32.4% from $18,683 (95%CI $17,747- $19,619 to $12,637 (CI $11,581-$13,693). This result is consistent with Hadley (2007) who found that an uninsured person who experiences a new chronic condition has greater difficulty obtaining recommended medical care. In such cases when their health remains compromised, these individuals can have more difficulty obtaining health insurance in the future which subsequently contributes to reduced labor force participation, lower productivity, and lower income.

Third, we found that even after controlling for chronic diseases as well as functional limitations in addition to education, age and marital status, race continues to be a significant predictor of personal income. Compared to White/Non-Hispanic whose personal income declined by 6.9% after adjustment, mean personal income declined by 18.2% for Black/Non-Hispanic and 31.0% for Hispanic. Further research needs to be conducted to understand the reason(s) for the racial disparity in income.

Despite what we believe are novel findings, this study has some limitations. The MEPS is a household survey based on self-report and has the potential for recall bias which may be important in reporting chronic diseases which depend on having been told the individual has a certain condition by a health care provider. Similarly functional limitations are subject to self-report and do not depend on a provider’s diagnosis but the opinion of the responder. Second, the study is limited to data from a single year. We recognize that economic and other societal conditions affecting personal income change over time so that a longitudinal study would be preferable (Martin et al., 2008). We have attempted to control for potential bias in this study by using the Heckman method.

## 5. Conclusions

This study provides new insights into the relationship between chronic diseases, functional limitations and personal income. Although many chronic diseases impact personal income via their associated functional limitations, some functional limitations have a greater effect than others. Depression, social/cognitive limitations, race, gender, marital status, education, insurance status and health status are independently associated with reductions in personal income in U.S. adults with chronic diseases. The findings of this study suggest a need to determine the specific limitations associated with common chronic conditions and identify appropriate compensatory strategies to reduce their impact.
